# Genotype by environment analysis on multi-canopy cropping system towards harvest in soybean

**DOI:** 10.1016/j.heliyon.2023.e16488

**Published:** 2023-05-26

**Authors:** Rahmadina, Isnaini Nurwahyuni, Elimasni, Diana Sofiah Hanafiah

**Affiliations:** a Department of Biology, Faculty of Mathematics and Natural Sciences, Universitas Sumatera Utara, Medan, North Sumatra, 20155, Indonesia; b Department of Agriculture, Faculty of Agriculture, Universitas Sumatera Utara, Medan, North Sumatra, 20154, Indonesia

**Keywords:** Factorial randomized block, Yield, Monoculture, Multi-canopy, Soybean

## Abstract

The multi-canopy cropping system is a new way to increase the number of soybeans grown. It is based on the idea of vertical agriculture. Short and tall plants are grown on the same hill in this method. The tall plants form a canopy, so the vertical space can be used to grow crops. The study aimed to determine how breeding programs could be used to create rice varieties for a multi-canopy cropping system. The tests were done in the dry and wet seasons at the Universitas Sumatera Utara in Medan, Indonesia. For plant height, number of leaves, number of branches, and number of pods, the genotype x canopy system interaction effect was significant. Over the two seasons, the average yield of the multi-canopy cropping system was 6.61 t ha^−1^ compared to 5.59 t ha^−1^ for the monoculture. The average yield of seven genotypes in two cropping systems, monoculture and multi-canopy, was 5.59 t ha^−1^ and 6.62 t ha^−1^. The mean average agronomic traits over monoculture and multi-canopy plant height, number of leaves, number of branches, and number of pods were 67.63 cm, 28.83, 8.00, and the number of pods 154.42. The AMMI analysis shows most of the differences between genotype x environment. The first group is made up of the environment during the dry season and the wet season. The mean net assimilation rate of soybean genotypes under multi-canopy and monoculture systems monoculture and multi-canopy was 1.81 μg cm^2^ d^−1^ and 2.87 μg cm^2^ d^−1^. The tall and short genotypes in multi-canopy have the highest yield, so they could be used to breed rice varieties that do well in multi-canopy.

## 1 Introduction

Beans were an important source of protein throughout Old and New World history and still is today. Beans, including soybean (*Glycine* max L.), are the seed of several plants in the family Fabaceae, used as vegetables for human or animal food. They can be cooked in many different ways, including boiling, frying, and baking, and are used in many traditional dishes worldwide [[[1], [2], [3]]. Soybean came from East Asia and was first domesticated around 5000 years ago [4]. A few Asian landraces were brought to North America in the first half of the 20th century. These landraces became the genetic base of North American cultivars, which went through eight decades of intense breeding to create the elite cultivars now grown widely in North America and elsewhere [5]. Crop scientists and soybean farmers have worked together over the past few decades to raise the world's soybean production from 155.1 million tons in 1999 to 284 million tons in 2013 [6]. Other than rice and corn, which have been produced for a long time [[7], [8], [9]], soybean (*Glycine* max L.) is a food staple [10]. Soybeans make up about 55% of the world's oilseed production, and over the last 10 years, their production has grown at an average rate of over 5% per year. Soybean is the only oilseed crop comprising 53% of global production. Other crops like rapeseed, cotton, and peanuts comprise 15, 10, and 9%, respectively. The United States grows soybeans on most land and produces about 32% of the world's soybeans. Brazil, Argentina, China, and India follow that order [11].

To improve the grow soybeans is necessary to find alternatives to monoculture, and this study offers the possibility of a multi-canopy cropping system. A multi-canopy system plants two types of soybeans at different heights on the same hill. This method of growing uses both tall and short genotypes. The difference in height between tall and short plants makes a layered panicle so that the vertical harvest space can be used. Even though studies have been done on cultivar mixtures, we see the multi-canopy system as a new way to increase soybean yield. Cultivar mixtures are combinations of seeds from different genotypes grown together in the same field. Studies have shown that cultivar mixtures increase soybean yields compared to monoculture [12,13]. Cultivar mixture is one of the most common ways to keep pathogens from hurting crop plants [[14], [15], [16]].

The competition between plants with different genotypes is a vital thing to think about when mixing cultivars [[17], [18], [19]]. Plants compete because they have traits that grab resources and take over the growth space [20,21]. In a multi-canopy system, the root zone of two different genotypes may be the same, and the taller genotype may shade the shorter genotype. The competition between tall and short rice genotypes to soak up sunlight is another effect of the cultivar mixture cropping system. Shade stress can cause super-hybrid rice biomass to lose up to 29.9% of its dry weight [22].

Multy-canopy cropping systems will significantly affect plant growth. Compared to a monoculture cropping system, multi-canopy by combining plant types, namely tall and short genotypes, has a lot of influence. Furthermore, observations were made of genotypes by the environment, such as season and canopy interactions, to provide reasonable cropping system technique conclusions in this study. This study aims to determine the potential for planting varieties in the multi-canopy cropping system in soybean.

## 2 Materials and methods

### 2.1 Plant material and experimental sites

The research will be conducted from December 2021 to July 2022 on farmer land on Jl. Ikahi, Kecamatan Medan Selayang, Kota Medan. Plant morphology analysis was conducted at the Biology Laboratory, FMIPA, Universitas Sumatera Utara, Medan.

### 2.2 Experimental design

The multi-canopy system experiment followed by Widyastuti et al. with the research design used was a 3-factorial randomized block design (RAK), namely soybean cultivar variation, spacing, and pruning with 3 replications through a multi-canopy cropping system on black soybean cultivars [17]. This planting was carried out at a spacing of 20 × 20 cm. The final result of this research is to see the best cultivar from several cultivars with pruning treatment and tight spacing through a multi-canopy planting system. The tall genotype, namely soybean plants with high plant height, symbolized USUK2P1J1, USUK2P1J2, USUK2P2J1, and USUK2P2J2. The short genotype, namely soybean plants with low plant height, symbolized USUK4P1J1, USUK4P1J2, USUK4P2J1, and USUK4P2J2.

### 2.3 Statistical analysis

**Fig. 1:**
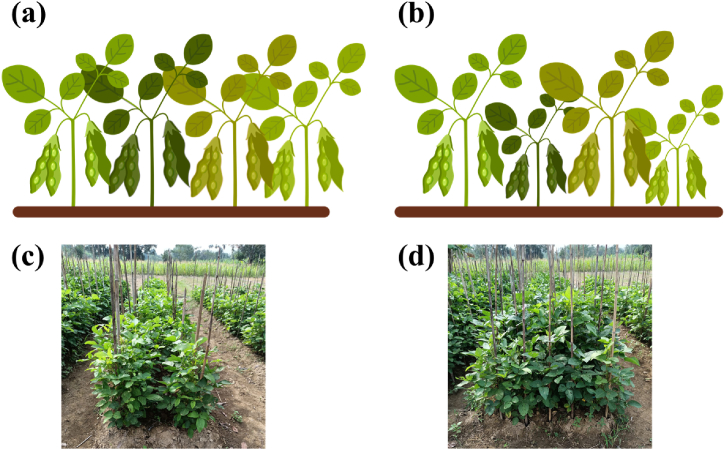
Illustration of (a) monoculture and (b) multi-canopy cropping systems in soybean, and it appeared in the field (c and d, respectively).

A plot's tall and short genotypes were manually separated in the multi-canopy system, and the tall and short genotypes were then harvested. The harvest times were comparable for both tall and short genotypes, monoculture, and multiple-canopy systems. The moisture content of each Genotype grain height was then measured before the grains were weighed. The multi-canopy yield was calculated. Each plot's plants were sampled to harvest to quantify the agronomic characteristics of the genotypes of short plants. Plant samples from four hills were taken for each area. The illustration of the monoculture and multi-canopy cropping system in soybean can be seen in Fig. 1.

Rstudio was used to do statistical analyses. For comparing the means of genotypes, the Least Significant Differences (LSD) at the 0.05 level were calculated. The Malosetti et al. method determined the average response of all genotypes in each environment to each environmental index [23]. Each trait's average difference between monoculture and multi-canopy was calculated for each genotype. Based on these values, the correlation between traits was then calculated. Using Rstudio, additive main effects and multiplicative interactions (AMMI) analysis were done to determine how stable the genotype's plant height and yield were. Data on maximum humidity, minimum humidity, maximum temperature, minimum temperature, and rainfall in the dry and wet seasons are obtained at Badan Meteorologi, Klimatologi, dan Geofisika (BMKG).

## 3 Results and discussion

### 3.1 Analysis and comparison of yields for all cropping systems

From December 2021 to July 2022, the study was carried out at the Universitas Sumatera Utara Experimental Station in Medan, Indonesia, as seen in Table 1. The experiments used two environments, the dry season and the wet season. The maximum humidity, minimum humidity, maximum temperature, minimum temperature, and rainfall were 98.75%, 57.5%, 33.5 °C, 21.25 °C, and 22.5 nm, respectively, in the dry season (April 2022–July 2022), while it was 99.00%, 63.75%, 33.50 °C, 21.25 °C, and 29.5 nm, respectively, in the wet season (December 2021–March 2022).

**Table 1:** Weather data in the dry season and wet season in the experimental station.

Month	Maximum humidity (%)	Minimum humidity (%)	Maximum temperature (^o^C)	Minimum temperature (^o^C)	Rainfall (mm)
Dry season					
April	98	60	34	21	24
May	100	55	33	20	21
June	99	57	33	23	22
July	98	58	34	21	23
Wet season					
December	99	62	33	22	28
January	100	64	34	21	28
February	99	64	34	20	34
March	98	65	33	22	28

The combined analysis of variance revealed that the effects of the multi-canopy and monoculture systems were significant (p < 0.05) for all traits, as seen in Table 2. The season, canopy, and genotype impact were significant for all traits. The genotype by season (G × S) interaction significantly affected plant height, and the number of branches at significant (p < 0.05) was 3.38 and 1.04. All traits were significantly affected by the genotype by the canopy (G × C) interaction effect at significant (p < 0.01), except the number of branches was 22.04, 42.67, and 6112.04. G × C interaction effect at significant (p < 0.05) affected number of branches was 0.38. Except for the number of leaves, all traits were significantly impacted by the genotype by canopy by season (GxCxS) interaction at significant (p < 0.01) was 3.38, 2.04, and 165.38.

**Table 2:** Mean squares from the combined analysis of variance for quantitative traits.

Sources of variation	df♦	Traits			
		Plant height	number of leaves	number of branches	number of pods
Season (S)	1	26.04**	8.17**	9.37**	145.04**
Canopy (C)†	1	165.38**	294.00**	45.38**	53110.04**
Genotype (G)	23	77.04**	88.17**	2.04**	17013.38**
G × S	23	3.38*	0.17 ns	1.04*	0.04 ns
G × C	23	22.04**	42.67**	0.38*	6112.04**
S × C	23	7.04*	6.00**	0.04 ns	408.38**
G × C × S	23	3.38*	2.67 ns	2.04**	165.38**
CV, %		9.08	10.92	2.33	14.90

† Canopy refers to the cropping systems (monoculture and multi-canopy). ♦ df: Degrees of freedom. * Significant at p < 0.05; ** Significant at p < 0.01; ns Not significant.

The average yield of seven genotypes in two cropping systems can be seen in Table 3. The average yield produced in a multi-canopy system is 6.62 t ha^−1^. As a result, the yield on multi-canopy is higher than that of the monoculture system, which is only 5.59 t ha^−1^. It shows that the multi-canopy method provides the potential to increase yields on soybean. Because the short and tall genotypes were both planted on the same hill, the tall plant affected the growth and yield of the short plants. In the multi-canopy system, the yield of short genotypes was higher than in the monoculture at 5.85 t ha^−1^. It shows that the tall genotype competed with the short genotype for resources. Under the same agronomic and cultural conditions, with two seedlings per hill for monoculture and multi-canopy, the genotypes tested showed different yield loss amounts. It means that there are different genes or ways to control these traits. So, making a breeding program and choosing genotypes for multi-canopy is a good idea.

**Table 3:** The average yield of seven genotypes in two cropping systems.

Genotype	Monoculture (t ha^−1^)	Multi-canopy (t ha^−1^)
Tall genotype		
USUK2P1J1	5.50 ± 0.34	6.66 ± 0.29
USUK2P1J2	5.44 ± 0.12	6.04 ± 0.27
USUK2P2J1	5.18 ± 0.06	6.32 ± 0.11
USUK2P2J2	5.19 ± 0.15	6.18 ± 0.12
Short genotype		
USUK4P1J1	5.73 ± 0.53	6.63 ± 0.07
USUK4P1J2	6.09 ± 0.10	6.85 ± 0.19
USUK4P2J1	5.95 ± 0.22	7.18 ± 0.17
USUK4P2J2	5.66 ± 0.04	7.05 ± 0.31
Mean	5.59	6.62
Standard derivation	0.33	0.41
CV, %	6.56	6.34

Complementary and compensation can yield more cultivar mixtures [24]. Even though the practical aspects of a multi-canopy system are different from a typical cultivar mixture, yield benefits from cultivar mixtures have been seen in many crops [25,26]. A meta-analysis of 91 studies and more than 3600 observations on cultivar mixtures showed that cultivar mixtures increased yield by 2.2% compared to monoculture [27]. A recent study showed that a cultivar mixture yields more wheat than a monoculture [28]. Other studies showed that a cultivar mixture could increase yield and keep yield stable under certain stress conditions. Several studies have also shown higher multi-canopy yields compared to monocultures. Widyastuti et al. shows higher multi-canopy yield than monoculture systems in rice [17]. Adham et al. show corn's higher multi-canopy yield monoculture system [29]. Ahakpaz et al. also shows a higher multi-canopy yield than the monoculture system in barley [30].

### 3.2 Effects of a multi-canopy system on agronomic traits and yield components

In the multi-canopy system, the averages of several short genotype traits in the monoculture system, as seen in Table 4. In the multi-canopy system, the plant height, number of leaves, number of branches, and pods of short genotypes all went up. It made ample space between the tall and short genotypes in a multi-canopy. Because a wide stratified gap separates the tall and short genotypes, there is little competition for sunlight and carbon dioxide. It means that photosynthesis, which makes it assimilate to send to other plant organs, works best [31]. Even though it has a wide stratified gap, it makes the number of pods, which makes people think that multi-canopy plants might be able to increase yield potential. Mixed culture could slow stem cell growth at the start of the plant's life cycle because root growth would be focused on getting enough resources in competitive conditions [32]. The adaptability of a genotype depends on its genetic makeup, cultivation methods, and growing conditions [33].

**Table 4:** Average agronomic traits over monoculture and multi-canopy.

Genotype	Plant height (cm)	number of leaves	number of branches	number of pods
Tall genotype				
USUK2P1J1	65.67 ± 1.53	22.00 ± 1.00	7.67 ± 0.58	94.33 ± 3.21
USUK2P1J2	69.67 ± 0.58	29.33 ± 0.58	6.33 ± 0.58	121.00 ± 1.00
USUK2P2J1	67.00 ± 1.00	33.33 ± 1.53	10.00 ± 0.00	170.00 ± 1.00
USUK2P2J2	68.00 ± 1.00	28.00 ± 1.00	9.33 ± 0.58	234.33 ± 15.28
Short genotype				
USUK4P1J1	61.00 ± 1.00	22.67 ± 1.53	5.33 ± 0.58	103.00 ± 2.00
USUK4P1J2	68.00 ± 1.00	28.33 ± 1.53	6.00 ± 0.00	119.00 ± 1.00
USUK4P2J1	70.00 ± 1.00	30.67 ± 0.58	9.00 ± 0.00	151.67 ± 3.79
USUK4P2J2	71.67 ± 0.58	32.33 ± 1.53	10.33 ± 0.58	242.00 ± 2.65
Mean	67.63	28.33	8.00	154.42
Standard derivation	3.23	4.08	1.89	54.91
CV, %	4.77	14.41	23.60	35.56

**Fig. 2:**
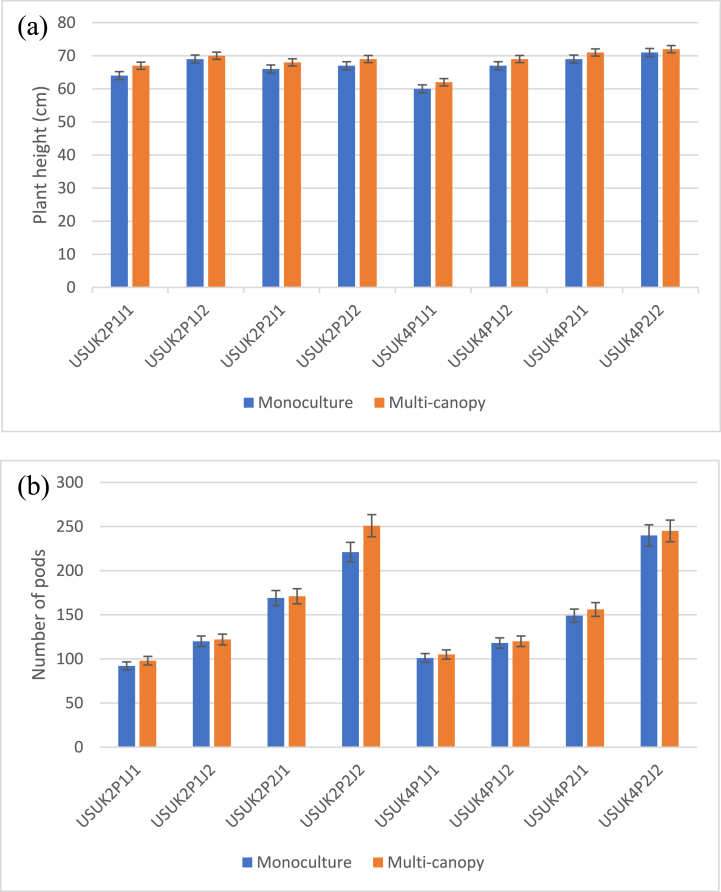
Changes in the (a) plant height and (b) number of soybean pods under monoculture and multi-canopy cultivation.

Fig. 2 shows how the multi-canopy cropping system changed these traits of genotypes. It shows that the plant height of the genotypes increased from monoculture to multi-canopy, while the plant height of the other genotypes did not change, likewise on the number of pods. The decrease or increase in these traits could be caused by light interception competition, which happens when tall genotypes shade short genotypes with their multiple canopies, or by stress on root growth space, which happens when two genotypes are planted in the same planting hole. According to Fiorucci et al., when *Arabidopsis thaliana* is grown in a mixed culture system with whole light, neighbouring plants can reflect much far-red light onto receptor plants the ratio of red to far-red light decreases [34]. It causes changes in the plant's appearance, such as longer stems, internodes, petioles, raised leaves, less branching, and faster flowering.

Phenotypic plasticity is the ability of genes to change in response to different environments [35]. Nguyen et al. show that plant architecture, such as tillering pattern, plant height, leaf shape and arrangement, and panicle shape, are necessary for yield [36]. Burgess et al. show that taller plants have a competitive advantage because longer, upright leaves give them better access to light [37]. At the same time, they shade the canopy below them. Abichou et al. show that competition changes the tillers' arrangement, the leaves size, and where the leaves are on the plant when the plant spacing is changed [38].

### 3.3 AMMI analysis

**Fig. 3:**
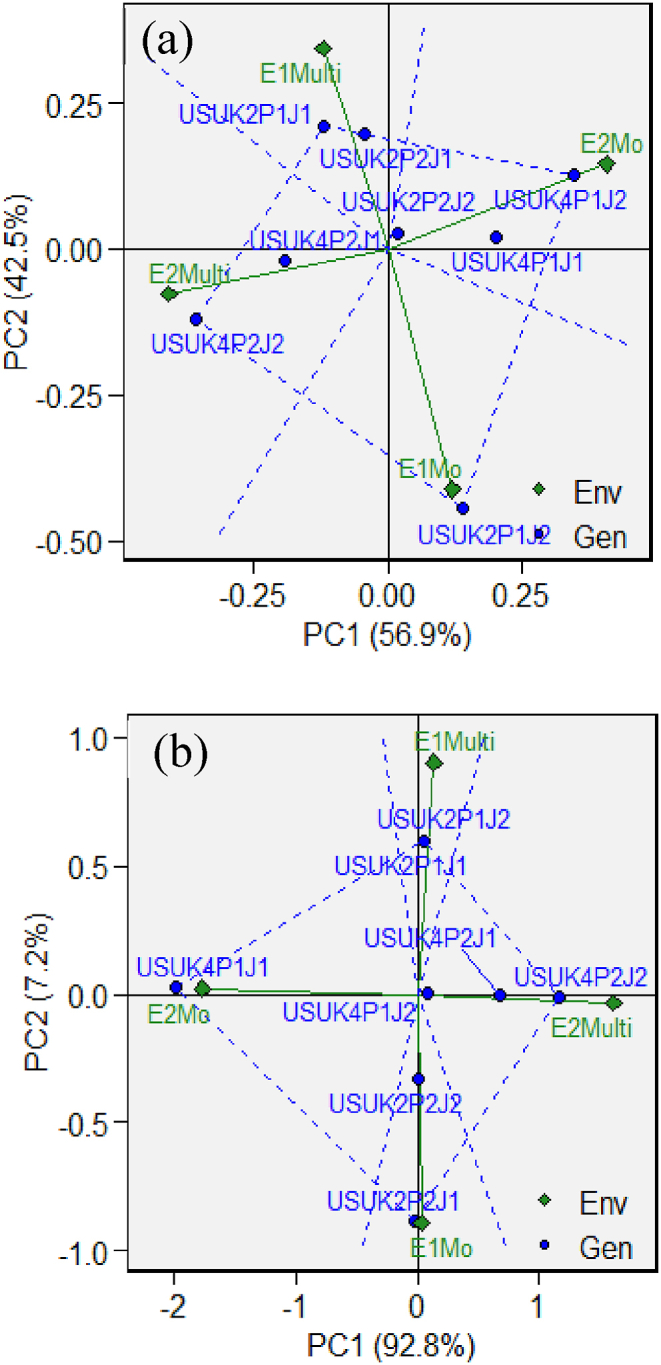
Ammi biplot of (a) plant height and (b) yield of soybean showing groups of environments and genotypes. E1Multi, multi-canopy in the dry season; E2Multi, multi-canopy in the wet season; E1Mo, monoculture in the dry season; E2Mo, monoculture in the wet season.

The AMMI analysis shows that the first two principal components in Fig. 3 Can explain most of the differences between G x E. The first group is made up of the environment during the dry season and the wet season. The second group is made up of canopy systems during both the dry and wet seasons. Genotypes at an angle should have plant height taller and produce more in the environments of the sector. In the plant height section (Fig. 3a), it is found that the USUK2P1J1 genotype is the best, and it is found in a multi-canopy system. In the soybean yield section (Fig. 3b), the best genotype is also in USUK2P1J1 in multi-canopy systems. It shows that combining tall and short genotypes results in better plant height and yield.

### 3.4 Net assimilation rate

The net assimilation rate quantifies the capacity of photosynthesis to generate dry matter in plants. The relationship between leaf area and net absorption rate is close [39]. The broader the leaf, the greater the net absorption rate. The system with many canopies has more leaves than the monoculture. Additionally, it influences the net assimilation rate. The net assimilation rate is proportional to the leaf area produced during a given time. Limiting leaf growth will reduce the leaf area's ability to absorb light, reducing the generated seed output [40]. Table 5 shows the net assimilation rate revealing that bean plants benefit from multi-canopy. According to research by Evers et al., multi-canopy gives a comparable net assimilation rate to traditional cropping systems [41].

**Table 5:** Net assimilation rate of soybean genotypes under multi-canopy system and monoculture systems.

Genotype	Monoculture (μg cm^2^ d^−1^)	Multi-canopy (μg cm^2^ d^−1^)
Tall genotype		
USUK2P1J1	1.86 ± 0.02	2.52 ± 0.59
USUK2P1J2	1.76 ± 0.02	3.10 ± 0.02
USUK2P2J1	2.17 ± 0.07	3.32 ± 0.01
USUK2P2J2	0.94 ± 0.02	2.10 ± 0.02
Short genotype		
USUK4P1J1	1.50 ± 0.06	2.40 ± 0.08
USUK4P1J2	1.64 ± 0.01	2.50 ± 0.07
USUK4P2J1	2.51 ± 0.05	3.25 ± 0.11
USUK4P2J2	2.12 ± 0.03	3.73 ± 0.07
Mean	1.81	2.87
Standard derivation	0.45	0.56
CV, %	24.88	19.40

## 4 Conclusions

For plant height, number of leaves, number of branches, and number of pods, the G x C system interaction effect was significant (p < 0.05). Over the two seasons, the average yield of the multi-canopy cropping system was 6.61 t ha^−1^ compared to 5.59 t ha^−1^ for the monoculture. The tall and short genotypes in multi-canopy have the highest yield, so they could be used to breed rice varieties that do well in multi-canopy. The average yield of seven genotypes in two cropping systems, monoculture and multi-canopy, was 5.59 t ha^−1^ and 6.62 t ha^−1^. The mean average agronomic traits over monoculture and multi-canopy plant height, number of leaves, number of branches, and number of pods were 67.63 cm, 28.83, 8.00, and the number of pods 154.42. The AMMI analysis shows most of the differences between G x E. The first group is made up of the environment during the dry season and the wet season. The mean net assimilation rate of soybean genotypes under multi-canopy and monoculture systems monoculture and multi-canopy was 1.81 μg cm^2^ d^−1^ and 2.87 μg cm^2^ d^−1^.

## Author contribution statement

Rahmadina: Conceived and designed the experiments; Performed the experiments; Analyzed and interpreted the data; Contributed reagents, materials, analysis tools or data; Wrote the paper.

Isnaini Nurwahyuni: Conceived and designed the experiments; Analyzed and interpreted the data; Contributed reagents, materials, analysis tools or data; Wrote the paper.

Elimasni and Diana Sofiah Hanafiah: Analyzed and interpreted the data; Wrote the paper.

## Data availability statement

Data will be made available on request.

## Additional information

No additional information is available for this paper.

## Declaration of competing interest

The authors declare that they have no known competing financial interests or personal relationships that could have appeared to influence the work reported in this paper.
